# Immunomodulatory Therapy Reduces the Severity of Placental Lesions in Chronic Histiocytic Intervillositis

**DOI:** 10.3389/fmed.2021.753220

**Published:** 2021-10-18

**Authors:** Chloe A. Brady, Charlotte Williams, Gauri Batra, Elaine Church, Clare L. Tower, Ian P. Crocker, Alexander E. P. Heazell

**Affiliations:** ^1^Tommy's Maternal and Fetal Health Research Centre, St Mary's Hospital, The University of Manchester, Manchester, United Kingdom; ^2^Exeter Medical School, University of Exeter, Exeter, United Kingdom; ^3^Pediatric Histopathology, Central Manchester University Hospitals National Health Service Foundation Trust, Manchester, United Kingdom; ^4^Saint Mary's Hospital Managed Clinical Maternity Service, Manchester Academic Health Science Centre, Manchester, United Kingdom

**Keywords:** placental histopathology, prednisolone, hydroxychloroquine, stillbirth, treatment, miscarriage, outcomes

## Abstract

Chronic histiocytic intervillositis (CHI) is a rare, but highly recurrent inflammatory placental lesion wherein maternal macrophages infiltrate the intervillous space. Pregnancies with CHI are at high risk of fetal growth restriction, miscarriage or stillbirth. Presently, the diagnosis can only be made after histopathological examination of the placenta. Given its proposed immunological etiology, current treatments include aspirin, heparin, and immunomodulatory agents. However, the rationale for these medications is largely based upon small case series and reports as there is a lack of larger studies investigating treatment efficacy. Therefore, this study sought to determine whether inclusion of immunomodulatory medications was effective at reducing the severity of lesions and improving pregnancy outcomes in subsequent pregnancies. Thirty-three women with a history of CHI in at least one pregnancy (index case) were identified retrospectively through medical records. Twenty-eight participants presented with a first subsequent pregnancy and a further 11 with a second subsequent pregnancy at a specialist clinic for pregnancy after loss. Data on maternal demographics, medical history, medication, pregnancy outcome, and placental pathology was collected and compared between pregnancies. Twenty-seven (69%) subsequent pregnancies were treated with at least one or both of prednisolone and hydroxychloroquine. Inclusion of at least one immunomodulatory agent in treatment regimen resulted in an almost 25% increase in overall livebirth rate (61.5 vs. 86.2%). In women treated with immunomodulatory medication a greater proportion of placentas had reduced severity of lesions compared to those treated without (86.7 vs. 33.3%, respectively). A reduction in CHI severity was associated with a 62.3% improvement in livebirth rate compared to those where severity remained unchanged in relation to the index case. These data provide preliminary evidence that the use of immunomodulatory medication in the management of CHI improves histopathological lesions and the chance of livebirth in subsequent pregnancies. Due to CHI's rarity and ethical and feasibility issues, randomized controlled trials in affected women are challenging to conduct. As a result, collaboration between centers is required in future to increase study sample sizes and elucidate the mechanisms of hydroxychloroquine and prednisolone in reducing pathology.

## Introduction

Chronic histiocytic intervillositis (CHI), also known as chronic intervillositis or chronic intervillositis of unknown etiology (1), is a pregnancy disorder strongly associated with fetal growth restriction, miscarriage, stillbirth and neonatal death (2–5). Estimated to affect 6 in every 10,000 pregnancies over 12 weeks' gestation, CHI is characterized by maternal macrophage infiltration into the intervillous space of the placenta and has a 25–100% risk of recurrence in subsequent pregnancies (1, 3, 6, 7). Many cases also exhibit marked perivillous and/or intervillous fibrin deposition and trophoblast necrosis (8). Due to its asymptomatic nature and a lack of reliable associated biomarkers, currently a diagnosis of CHI can only be made following delivery by histopathological examination of the placenta. Management of CHI is further complicated by a lack of standardized treatment options proven to prevent recurrence.

Owing to the presence of maternal macrophages and the reported increased incidence in women with autoimmune disease, CHI has been hypothesized to be a disorder of failed maternal-fetal tolerance and excessive inflammation (9). On this basis and due to the presence of intervillous and perivillous fibrin, current treatments include thromboprophylactic agents such as aspirin and low-molecular-weight heparin (LMWH) as well as those aimed at suppressing inflammation e.g., corticosteroids and hydroxychloroquine (9). A systematic review of six observational studies conducted in 2010 found no significant improvement in pregnancy outcome with aspirin and LMWH alone. Though a growing number of case reports detail use of immunomodulatory agents such as prednisolone and hydroxychloroquine (9, 10), there remains a striking lack of larger studies supporting the efficacy of any treatment regime in reducing severity of CHI and improving pregnancy outcomes. Notably, a prior case series has highlighted that women with worse obstetric histories tend to be prescribed more therapeutic agents, despite their unproven efficacy (9). Due to the high rate of recurrence of CHI and severe consequences of the disorder, studies are urgently needed to determine effective therapies.

By retrospectively identifying women with a previous diagnosis of CHI, we aimed to investigate pregnancy outcomes and placental pathology in subsequent pregnancies referred to a specialist service following poor perinatal outcome, stillbirth or neonatal death. We hypothesized, due to the immunological nature of CHI, that treatment regimens where immunomodulatory agents were included would decrease the severity of the condition and consequently improve the chance of livebirth.

## Materials and Methods

### Participant Recruitment and Data Collection

Women with a previous histopathological diagnosis of CHI between 2009 and 2021 were identified retrospectively from medical records at Manchester University NHS Foundation Trust, UK. The majority of these cases were identified after the death of a baby or late miscarriage when histopathological evaluation of the placenta is recommended practice. In other cases (following the birth of a live infant) the placenta is sent away for examination for a variety of clinical indications (e.g., FGR, fetal compromise at birth, placental abruption, and previous late pregnancy loss). CHI was diagnosed by a specialist perinatal pathologist in accordance with its initial description by Labarrere and Mullen as a placental lesion consisting of histiocytic (macrophage) infiltration into the intervillous space (8). Data on maternal demographics, medical history including results of tests for autoantibodies (lupus anticoagulant, anti-phospholipid, antinuclear, and anticardiolipin antibodies) and obstetric history were collected from the woman's case record. The first pregnancy diagnosed with CHI was classified as the “index” case, with data on any subsequent pregnancies recorded where applicable from retrospective medical records. Pregnancy outcomes consisted of liveborn and still living, liveborn at term and still living (>37 weeks' gestation), miscarriage (fetal death <24 weeks gestation, including spontaneous abortion), stillbirth (fetal death >24 weeks gestation), and termination of pregnancy for fetal anomaly (TOPFA). Cases of neonatal death (death of an infant within 28 days after birth) were also recorded. Fetal growth restriction (FGR) was defined as growth below the 3rd percentile, and small for gestational age (SGA) as between 3rd and 10th centile (11). Centiles were calculated for pregnancies >20 weeks' gestation using the GROW centile calculator, for cases where maternal demographics, pregnancy outcome and fetal sex were known (12).

The therapeutic agents used in our service evolved over time following publications from other researchers (3, 9, 10, 13) and following discussion with colleagues with expertise in lupus in pregnancy. Initially, pregnancies with CHI were managed using Aspirin and LMWH, but this evolved to a combination of Aspirin was given at a dose of 75–150 mg once a day, a prophylactic dose of LMWH (e.g., Tinzaparin 4,500iu) once a day, Hydroxychloroquine 200 mg twice a day and Prednisolone 20 mg once a day in the morning. Drug therapy was started from a viability scan at 6–7 weeks' gestation. Women underwent ultrasound assessment of uterine artery Doppler at 17 weeks' gestation, if this showed no abnormality then Prednisolone was reduced by 5 mg per week. If there was evidence of uterine artery notching or raised pulsatility index Prednisolone was continued at 20 mg and then uterine artery Doppler's were reassessed after 2 weeks. If there was no improvement by 21 weeks' gestation the Prednisolone was reduced at this stage. Women underwent regular ultrasound assessment of fetal growth, amniotic fluid volume and umbilical artery Doppler after 23 week's gestation at a minimum frequency of 3-weekly intervals.

Where available, placental histopathology reports were analyzed for detail on histopathological features including the presence of villitis, increased fibrin deposition and recurrence and severity of CHI. Change in severity of CHI in subsequent pregnancies was in comparison to the severity of the index case of CHI. Focal CHI without accompanying fibrin deposition was classified as “mild,” those with accompanying fibrin as “moderate,” and diffuse, high-grade lesions classified as “severe.”

Informed and written consent was obtained from all study participants and ethical approval granted by the NRES Committee North West—Greater Manchester West and NRES Committee London—City & East.

### Statistical Analysis

Statistical analysis was conducted using GraphPad Prism Version 9.1.0. Chi-square and Kruskal-Wallis tests were performed for categorical and continuous variables, respectively. For statistical analysis, first and second subsequent pregnancies were combined, and pregnancy outcomes divided into liveborn and still living or liveborn at term and still living vs. adverse outcome (TOPFA, miscarriage, stillbirth and neonatal death). Where sample number precluded analysis by Chi-square, Fisher's Exact test was performed instead. Statistical analysis of treatment regimen was performed by dividing participants into those which received immunomodulatory therapy (one or both of prednisolone and hydroxychloroquine) and those without (one or both of aspirin and heparin, or untreated).

## Results

### Participant Demographics

Thirty-three women with a history of at least one pregnancy affected by CHI between 2009 and 2021 were identified retrospectively from medical records. Twenty-eight women presented with a subsequent pregnancy and 11 of these with a further second subsequent pregnancy. Participant demographics, medical and obstetric history are shown in Table 1. Four Dichorionic-Diamniotic (DCDA) twin pregnancies were included in the study. Twenty-six women were White British (78.8%), with a smaller proportion of Asian (*n* = 6) and Black African women (*n* = 1). Four index CHI pregnancies (12.1%) were conceived using ART, compared to only one first subsequent pregnancy (3.6%) and no second subsequent pregnancies. Underlying autoimmune disease was present in four cases (12.1%), consisting of coeliac disease, hypothyroidism, autoimmune thrombocytopenia, and hypermobility syndrome, respectively. Seventeen women had been tested for antinuclear antibodies, three of which were positive (17.7%) and 25 and 22 women had testing for anti-cardiolipin antibodies and anti-phospholipid antibodies, respectively, none of which were positive. Of 26 women tested for lupus anticoagulant, one was positive (3.9%). Pre-existing hypertension was present in two women (6.1%).

**Table 1:** Demographics and obstetric and medical history of women with a diagnosis of chronic histiocytic intervillositis (CHI).

	**Index CHI**	**1st subsequent pregnancy**	**2nd subsequent pregnancy**
***N*** **participants**	33	28	11
***N*** **fetuses**	34	30	12
Maternal age (years)	32 (19–38)	34 (22–41)	37 (26–40)
BMI	26 (20–47)	27 (20–47)	27 (20–40)
**Ethnicity**			
White British	26 (78.8%)		
Asian	6 (18.2%)		
Black African	1 (3.0%)		
**Lifestyle**			
Smoker	3 (9.4%)	1 (3.6%)	0
Unknown	1		
Alcohol consumption	0		
Unknown	1		
**Obstetric history**			
Previous livebirths	0 (0–5)		
Previous losses	0 (0–4)		
Primigravida	11 (33.3%)		
ART pregnancy	4 (12.1%)	1 (3.6%)	0
Twin pregnancy	1 (3.0%)	2 (7.1%)	1 (9.1%)
**Medical history**			
Autoimmune disease	4 (12.1%)		
Pre-existing hypertension	2 (6.1%)		
Anti-nuclear antibodies	3 (17.7%)		
Untested	16		
Lupus anticoagulant	1 (3.9%)		
Untested	7		

*Index pregnancy was defined as a participant's first pregnancy diagnosed with CHI by placental histopathological examination following poor outcome. Subsequent pregnancies refer to those following diagnosis. Continuous variables are presented as median (range) and categorical variables N (percentage). ART, assisted reproductive technology; BMI, body mass index*.

### Pregnancy Outcomes

Outcomes of index pregnancies diagnosed with CHI and subsequent pregnancies are listed in Table 2. The proportion of infants born by Cesarean section increased from 27.3% (6/33) in index pregnancies to 59.1% (13/28) and 45.5% (5/11) in first and second subsequent pregnancies, respectively, though this was not statistically significant. Fetal sex did not differ significantly across pregnancies, and in two subsequent pregnancies fetal sex had not been determined due to loss early in gestation.

**Table 2:** Pregnancy outcomes of index and subsequent pregnancies in women with a diagnosis of chronic histiocytic intervillositis (CHI).

	**Index CHI**	**1st subsequent pregnancy**	**2nd subsequent pregnancy**
***N*** **participants**	33	28	11
***N*** **fetuses**	34	30	12
Cesarean section (>24 weeks)	6 (27.3%)	13 (59.1%)	5 (45.5%)
Male fetus	13 (38.2%)	5 (53.6%)	7 (58.3%)
Unknown fetal sex	0	2	0
**Pregnancy outcome**			
Liveborn and still living	4 (11.8%)	21 (70.0%)	12 (100%)
Stillbirth	16 (47.1%)	1 (3.3%)	0
Miscarriage	5 (14.7%)	6 (20.0%)	0
TOPFA	5 (14.7%)	1 (3.3%)	0
Neonatal death	4 (11.8%)	1 (3.3%)	0
Gestation at delivery (weeks)	26 (17–41)	37 (12–39)	38 (35–39)
Birthweight centile	2.8 (0–68.4)	22.8 (0.4–99.9)	23.2 (3.8–89.6)
**Complications**			
FGR <3rd centile	13 (52.0%)	3 (13.6%)	0
SGA 3rd−10th centile	5 (20.0%)	2 (9.1%)	1 (9.1%)
Preterm <37 weeks (% of livebirths)	6 (75.0%)	5 (22.7%)	3 (25.0%)
**Maternal comorbidities**			
Gestational diabetes	1 (3.0%)	0	0

*Index pregnancy was defined as a participant's first pregnancy diagnosed with CHI by placental histopathological examination following poor outcome. Subsequent pregnancies refer to those following diagnosis. Miscarriage was defined as fetal death <24 weeks gestation, and stillbirth as fetal death >24 weeks gestation. Neonatal death refers to the death of an infant within 28 days after birth. Continuous variables are presented as median (range) and categorical variables N (percentage). FGR, fetal growth restriction; SGA, small for gestational age; TOPFA, termination of pregnancy for fetal anomaly*.

The outcome of subsequent pregnancy/ies improved significantly, with 70% of first and 100% of second subsequent pregnancies resulting in a liveborn and still living infant, compared to 11.8% of index pregnancies (*p* < 0.0001). Gestation at delivery also increased significantly across pregnancies (*p* = 0.002), from a median of 26 weeks' gestation in index cases to 37 weeks' gestation in first subsequent (*p* = 0.04) and 38 weeks in second subsequent pregnancies (*p* = 0.002). Of infants who were liveborn, rates of neonatal death were significantly reduced from 50% (4/8) in index cases to 3.0% (1/33) of subsequent pregnancies (*p* = 0.003). TOPFA occurred in five index pregnancies (14.7%) and one first subsequent pregnancy (3.3%), after diagnosis of skeletal dysplasia (*n* = 1), severe FGR (*n* = 3), triploidy (*n* = 1) and neurological disorder (*n* = 1). In one case of stillbirth occurring in an index pregnancy, Trisomy 18 was diagnosed at post-mortem. For infants whose birthweight centiles could be calculated, rates of FGR <3rd centile decreased significantly between index and subsequent pregnancies from 52.0% (13/25) to 9.1% (3/33) (*p* = 0.0007). There were no significant differences in the incidence of SGA across pregnancies. Overall birthweight centiles increased significantly across pregnancies from a median of 2.8 in index cases to 22.8 and 23.2 in first and second subsequent pregnancies, respectively (*p* < 0.0001). In subsequent pregnancies, 23.5% of livebirths were preterm (8/34), significantly reduced from 75.0% (6/8) of index cases (*p* = 0.01). One participant was diagnosed with gestational diabetes in their index pregnancy (3.0%), and there were no cases of preeclampsia. Chorioamnionitis occurred in one index case (3.0%) alongside CHI, however since CHI recurred without infection in the participant's subsequent pregnancy this case was included in the study.

### Placental Pathology

Findings of placental histopathology are shown in Table 3. By design, all index pregnancies had an accompanying placental pathology report. The number of cases with histopathological examination of the placenta decreased to 76.7% (23/30) and 66.7% (8/12) in first and second subsequent pregnancies as a result of placentas not having been sent for histopathological examination. The incidence of CHI significantly decreased from index pregnancies, with an overall recurrence rate of 41.9% (13/31) in subsequent pregnancies (*p* < 0.0001). There were no significant differences in the incidence of villitis, increased fibrin deposition and placentas classified as small for gestational age. Pregnancies with concurrent CHI and villitis in the placenta exhibited no differences in outcome compared to those with CHI alone.

**Table 3:** Histopathology of placentas from index and subsequent pregnancies in women with chronic histiocytic intervillositis (CHI).

	**Index CHI**	**1st subsequent pregnancy**	**2nd subsequent pregnancy**
***N*** placentas	34	30	12
***N*** placentas with pathology reports	34 (100%)	23 (76.7%)	8 (66.7%)
CHI	34 (100%)	11 (47.8%)	2 (25.0%)
Chronic villitis	5 (14.7%)	6 (26.1%)	2 (25.0%)
Increased fibrin deposition	18 (52.9%)	7 (30.4%)	2 (25.0%)
Small for gestational age	7 (20.6%)	4 (17.4%)	1 (12.5%)

*Index pregnancy was defined as a participant's first pregnancy diagnosed with CHI by placental histopathological examination following poor outcome. Subsequent pregnancies refer to those following diagnosis. Variables are expressed as N (percentage)*.

**Figure 1:**
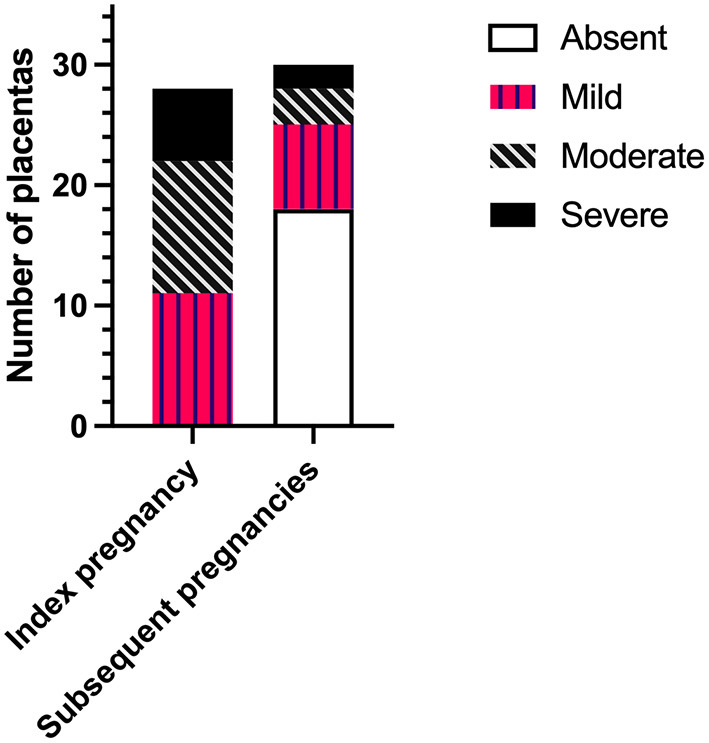
Severity of chronic histiocytic intervillositis (CHI) lesions in placentas from index and subsequent pregnancies according to pathologist's report. Index CHI refers to a participant's first pregnancy diagnosed with CHI by placental histopathological examination following poor outcome. Subsequent pregnancies refer to those following diagnosis.

The severity of CHI lesions in placentas from index and subsequent pregnancies as determined by histopathological examination is shown in Figure 1. Detail of CHI lesion severity was available for 28/34 (82.4%) and 30/31 (96.8%) placentas from index and subsequent pregnancies, respectively. Placentas from index cases exhibited mainly mild or moderate CHI [39.3% (11/28) and 39.3% (11/28)], followed by a smaller proportion of severe cases [21.4% (6/28). In comparison, CHI was absent in the majority of placentas from subsequent pregnancies (60% (18/30)], whilst 23.3% displayed mild lesions (7/30), 10.0% moderate (3/30), and 6.7% severe (2/30). Overall, severity of CHI was significantly reduced in subsequent pregnancies compared to index (*p* < 0.0001).

### Treatment Effect

**Figure 2:**
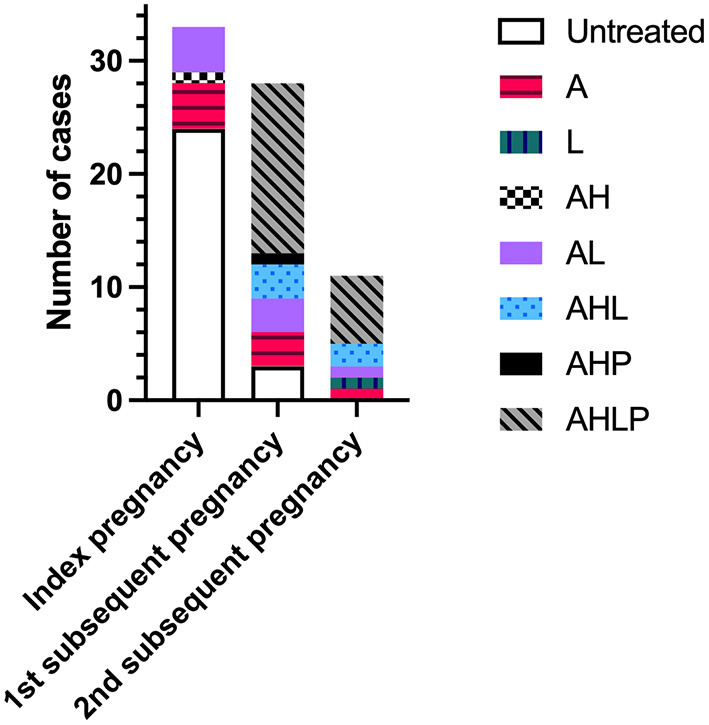
Treatment regimen across index pregnancies with chronic histiocytic intervillositis (CHI) and first and second subsequent pregnancies. Index pregnancy was defined as a participant's first pregnancy diagnosed with CHI by placental histopathological examination following poor outcome. Subsequent pregnancies refer to those following diagnosis. A, aspirin; H, hydroxychloroquine; L, low-molecular-weight heparin; P, prednisolone.

Treatment regimen across pregnancies is shown in Figure 2. The majority of index pregnancies had no medication (24/33, 72.7%), compared to 10.7% (3/28) of first subsequent pregnancies. No second subsequent pregnancies were untreated. In index pregnancies, the nine treated participants were on medication for pre-existing medical conditions or risk factors and took aspirin alone (*n* = 4), in combination with LMWH (*n* = 4), or hydroxychloroquine (*n* = 1). There was no significant difference in the livebirth rate of index pregnancies between women on medication and those without. In subsequent pregnancies, 69.2% (27/39) of all participants received immunomodulatory therapy with at least one or both of hydroxychloroquine or prednisolone, with the majority of participants 53.9% (21/39) receiving all four medications. All four women with a positive autoantibody screen were treated in their subsequent pregnancies (*n* = 5) with aspirin and LMWH (*n* = 1); aspirin, low-molecular-weight heparin and hydroxychloroquine (*n* = 1); aspirin, prednisolone, and hydroxychloroquine (*n* = 1) or a combination of all four medications (*n* = 2). These pregnancies all resulted in the birth of a live infant surviving past 28 days of life.

**Figure 3:**
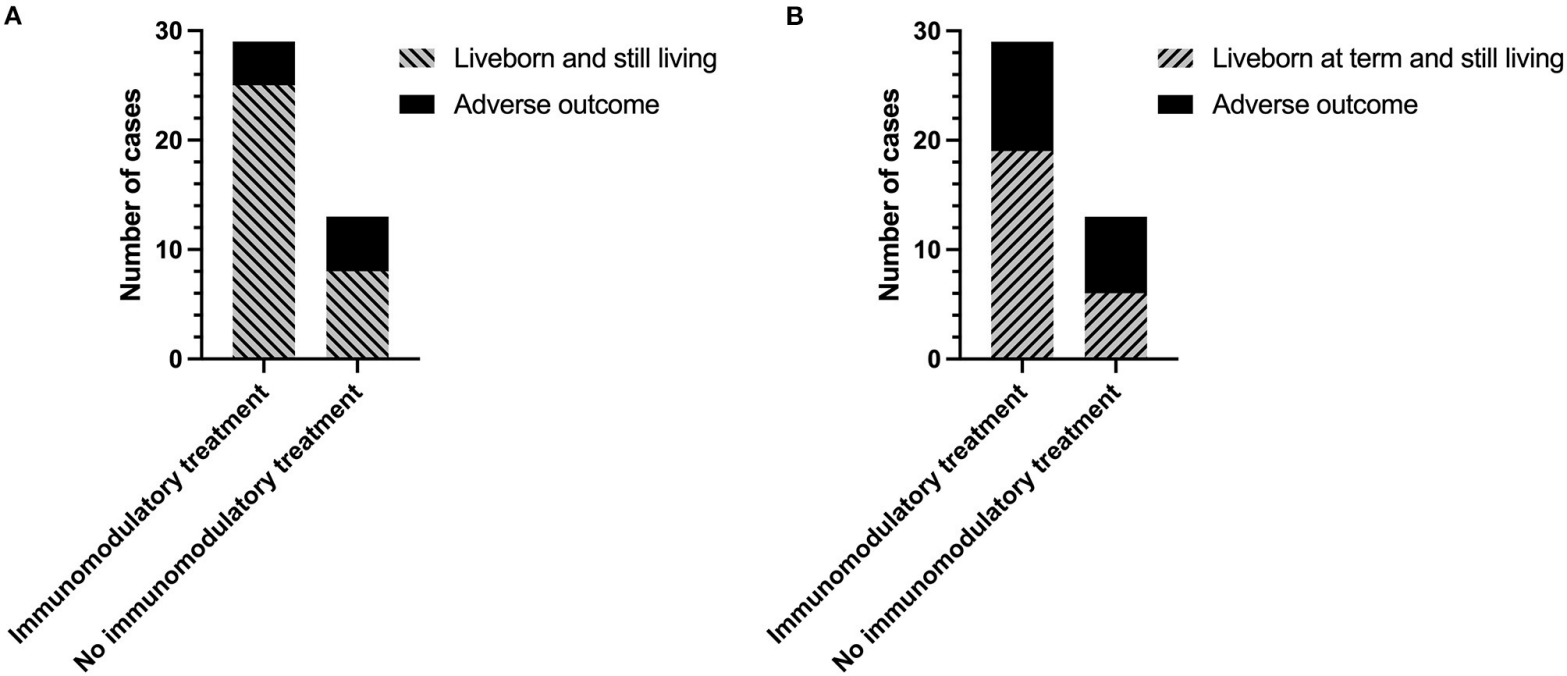
Outcomes of subsequent pregnancies in women with a previous diagnosis of chronic histiocytic intervillositis (CHI) following treatment with or without immunomodulators. **(A)** Number of pregnancies resulting in a liveborn and still living infant (including those born <37 weeks' gestation) or adverse outcome (miscarriage, termination of pregnancy, stillbirth, or neonatal death). **(B)** Number of pregnancies resulting in a liveborn and still living infant at term (>37 weeks), or adverse outcome (miscarriage, termination of pregnancy, stillbirth, preterm birth, or neonatal death) Immunomodulatory treatment refers to a regimen including one or both of prednisolone and hydroxychloroquine in combination with either aspirin, heparin, or both. Pregnancies without immunomodulatory treatment were untreated or received aspirin or heparin or both.

The overall rate of infants liveborn and still living (including infants born <37 weeks' gestation) was almost 25% higher in women receiving immunomodulatory treatment with one or both of hydroxychloroquine and prednisolone compared to those without [25/29 (86.2%) vs. 8/13 (61.5%)] (Figure 3A). However, this did not reach statistical significance due to the number of pregnancies included in the study (*p* = 0.11). Inclusion of immunomodulatory medication in treatment regimen also resulted in a 19% increase in the proportion of infants liveborn at term (>37 weeks) (Figure 3B) [65.5% (19/29) with immunomodulators vs. 46.2% (6/13) without], though again this was not statistically significant. There were no significant effects of immunomodulatory treatment on the incidence of FGR [8.33% (2/24) with vs. 12.5% (1/8) without], SGA [12.5% (3/24) with vs. 12.5% (1/8) without], or on overall birthweight centiles (median 20.9 with vs. 19.5 without).

**Figure 4:**
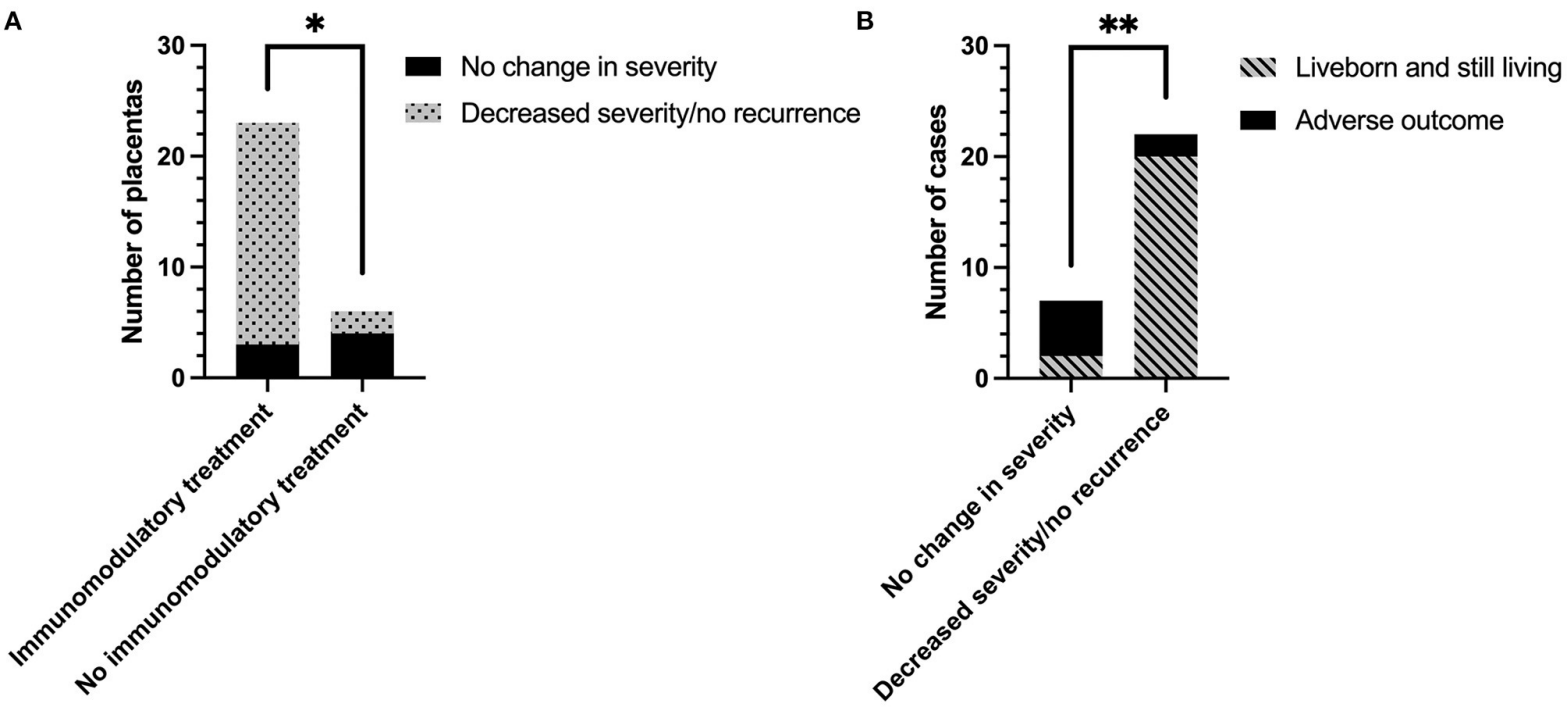
The effect of immunomodulatory medication in subsequent pregnancies after a diagnosis of chronic histiocytic intervillositis (CHI). **(A)** Change in CHI lesion severity in pregnancies treated with or without immunomodulatory medication, compared to the participant's first placenta diagnosed with CHI from a previous pregnancy. Severity of CHI was determined by pathologist's report. Immunomodulatory treatment refers to a regimen including one or both of prednisolone and hydroxychloroquine in combination with either aspirin, heparin, or both. Pregnancies without immunomodulatory treatment were untreated or received aspirin or heparin or both. **(B)** Rate of infants liveborn and still living (past 28 days of life) in subsequent pregnancies related to change in CHI severity. *p < 0.05; **p < 0.01.

Change in CHI severity following treatment regimen is shown in Figure 4A. 7/13 (53.8%) placentas from women treated without immunomodulatory medication were sent for histopathological examination, compared to 23/29 of those who were treated with it (82.8%). Change in severity of CHI in one subsequent pregnancy treated without immunomodulatory medication could not be determined due to a lack of detail on severity of the index case. The majority of remaining placentas from pregnancies treated without immunomodulatory medication exhibited no change in CHI severity compared to their index case [66.7% (4/6)]. In comparison, 86.7% (20/23) of cases treated with immunomodulators displayed a reduction in CHI severity or a lack of recurrence (*p* = 0.02). Decreased severity of CHI in subsequent pregnancies was associated with a 62.3% increase in livebirth rate compared to pregnancies where severity was unchanged [20/22 (90.9%) vs. 2/7 (28.6%), respectively] (*p* = 0.003; Figure 4B).

## Discussion

These data suggest that the use of one or both of prednisolone and hydroxychloroquine in the treatment of CHI resulted in a reduction of disease severity and a trend toward an increase in livebirth rate. Overall, a decrease in CHI severity was associated with a 62.3% reduction in pregnancy loss. These findings suggest that management of CHI in a specialist center for women pregnant after loss improves outcomes, with all second subsequent pregnancies resulting in the birth of a healthy infant.

### Strengths and Limitations

Previously, treatment of CHI has been informed by case reports and small studies detailing successful pregnancy outcome following the use of prednisolone and other immunomodulatory agents (10), due to a lack of randomized controlled trials informing management. Given CHI's rarity and ethical and feasibility issues surrounding recruitment of women with such poor obstetric histories, it is unlikely that there will be a study where treatment is compared to placebo or a combination of agents known to be ineffective (e.g., aspirin and LMWH alone). As such, case series prove invaluable in guiding the care of women with a history of CHI. To date, this series is the largest to investigate the efficacy of immunomodulatory medication in the treatment of CHI and proposes a standardized protocol of medication to increase the chance of positive outcome in subsequent pregnancies.

Through analysis of medical records, we have been able to follow women through subsequent pregnancies following diagnosis of CHI and formulate a well-characterized cohort of a rare condition. However, we recognize that women who decided to become pregnant or became pregnant could differ from all women who have CHI. The demographic characteristics of our sample were similar to those from other case series (5, 14), suggesting that this potential selection bias had not significantly altered the nature of the sample.

In previous investigations, the rate of recurrence of CHI and accompanying outcome in treated pregnancies has been relatively well-documented, though there is a paucity of evidence regarding improvement in lesion severity. For the majority of pregnancies we included, detail of lesion severity was available in placental pathology reports, allowing any change to be related to an individual's index case. To our knowledge, this is the first time such an approach has been taken in a case series of treated pregnancies following CHI. Here, it was shown that although overall recurrence of CHI in participants treated with immunomodulatory treatment did not differ significantly compared to those treated without, the severity of lesions was greatly reduced. This indicates that inclusion of disease severity as an outcome in future studies on CHI may provide better insight into treatment effect rather than simple recurrence rate alone.

Due to the retrospective nature of this study, there were certain variables for which data was not available within medical records. For instance, many women whose index case of CHI occurred less recently had not received screening for all autoantibodies as this was a more recent practice. Additionally, the range of antibodies tested was limited, which may explain the low incidence of autoantibodies in this cohort in comparison to studies which looked more specifically at antibody status and have reported incidences between 29 and 58% (9). Since this suggestion of an autoimmune component to CHI, it has become standard practice within our center to offer women an antibody screen following referral. In future, this will allow better characterization of any possible role pre-existing antibodies may have within CHI and may inform immunosuppressive treatment if underlying conditions are present.

Several subsequent pregnancies included were without an accompanying placental histopathology report as a direct result of having not been sent for examination. In all these cases, all respective pregnancies had resulted in a livebirth. This is suggestive that there may have been a bias toward reporting pathology only in pregnancies resulting in poor outcome. In addition, pathologists were informed of patient's medical history which may have influenced diagnosis. In response to decreased rates of pathological examination in subsequent pregnancies, the importance of histopathological examination following a history of CHI is becoming increasingly recognized amongst midwives and clinicians within our center. Consequently, placental histopathology is now a routine requirement and is anticipated to improve sample size for prospective studies.

### Clinical Context

Though the specific effects of prednisolone and hydroxychloroquine could not be individually determined here, our data is suggestive that inclusion of at least one of these medications in the treatment of CHI can significantly reduce the severity of macrophage infiltration into the intervillous space. Further to this, a reduction in CHI severity increased the likelihood of a live birth. This is in line with previous evidence that pregnancies where CHI is severe or diffuse in the placenta are more strongly associated with poor outcome (15). Pregnancy outcomes in CHI and severity following hydroxychloroquine use have not been well-documented previously, although a single study stated that four out of six pregnancies where it was included in treatment regimen resulted in a liveborn infant (9). Prednisolone has also in several case reports been associated with improved pregnancy outcome and reduced severity of both CHI and fibrin deposition compared to treatment with aspirin or heparin alone (2, 10, 16). Within our cohort, there was no significant difference in fibrin deposition between index and subsequent pregnancies, and the majority did not have an increase in fibrin noted in their pathology report. This is perhaps unsurprising as fibrin deposition has been classified previously as an accompanying feature which may or may not occur alongside CHI (1).

Whilst the exact cause of pathology in CHI is unknown, current evidence suggests that it is driven by excessive maternal inflammation. Inflammatory features characterized so far include the presence of partner-directed T lymphocytes and antibodies (17) and deposition of complement in the placenta (18). Our observation that administration of prednisolone and hydroxychloroquine aimed at suppressing the maternal immune response reduces CHI severity is therefore consistent with this hypothesis. The specific mechanism of hydroxychloroquine and prednisolone's anti-inflammatory effects in CHI are unknown, though in mouse and *in vitro* models of antiphospholipid syndrome, it has been suggested that hydroxychloroquine is able to reduce complement activation and antibody binding to the syncytiotrophoblast (19). In addition, hydroxychloroquine does not appear to have negative effects on placental explants and increases release of anti-inflammatory cytokine interleukin-10 (19). As complement deposition and excessive inflammation is characteristic of CHI, further investigation into the mechanisms of immunomodulatory therapy in the condition is warranted.

Evidence of CHI as an alloimmune condition has also provided rationale for the use of other immunomodulatory and immunosuppressive routes of treatment, including intravenous immunoglobulin (IVIG) therapy, tumor necrosis factor (TNF) antagonists, and tacrolimus (20). In a case report by Abdulghani et al. (13), IVIG was used following the failure of prednisolone to produce successful outcome in a previous pregnancy, and resulted in two subsequent healthy pregnancies. Histopathological examination of both placentas revealed that CHI had not recurred in either case. Similarly, use of TNF antagonist adalimumab was reported in the pregnancy of a woman with recurrent intervillositis and has been proposed as a possible agent in the prevention of recurrent miscarriage (21). Though these case reports provide anecdotal evidence hinting at the benefits of these agents, there is still a lack of larger studies to justify their use and as such much controversy remains.

Despite the lack of comparative studies where the efficacy of immunomodulatory medication in treating CHI has been specifically investigated, a livebirth rate of 66.7% in 21 women receiving various combinations of aspirin, heparin, prednisolone and hydroxychloroquine has previously been reported by Mekinian et al. (9). Conversely, in the only systematic review of intervention in CHI to date, treatment was suggested to correlate with worse outcome, with a reported livebirth rate of 30.8% (3). Here, livebirth rate with inclusion of one or both of hydroxychloroquine and prednisolone was markedly higher than both studies at 86.2%. Importantly, the systematic review did not include any studies wherein hydroxychloroquine was administered, and treatment regimen was less consistent than that used within this case series, both of which may be factors in the differences observed. As in the aforementioned studies, differences in livebirth rates within this cohort were not proven to be significantly different between treatment groups, likely due to limited sample size considering that data did show a trend toward statistical significance. Given that the only systematic review is now a decade old, it is probable that an updated study of the literature is required to better characterize treatment combinations and their effect on pregnancy outcome.

In subsequent pregnancies, gestational age at delivery, fetal growth, and rates of preterm birth improved greatly, though these effects could not be significantly attributed to immunomodulatory treatment. It is possible that this is due to limited numbers, or an effect of treatment using LMWH and aspirin. Adverse outcomes may also have been reduced as a consequence of increased fetal monitoring following specialist care after previous pregnancy loss. As there were insufficient numbers of untreated women and all participants had attended the specialist clinic, it was not possible to distinguish whether these outcomes were a result of intervention or treatment. Increased fetal monitoring is unable to influence fetal growth, but delivery via Cesarean section showed a trend toward increasing across subsequent pregnancies despite lacking statistical significance. Other centers have reported low levels of spontaneous labor in cases of CHI and early delivery of a liveborn infant has been detailed in a case report following observations of fetal growth plateau (9, 10). Therefore, it is possible that electing for early delivery may have had a beneficial effect in select cases.

Recurrence rates of CHI in subsequent pregnancies vary widely between studies and have been reported between 25 and 100% (1, 3, 7). Within this cohort, recurrence of CHI was 41.9%, though the majority of pregnancies were treated which in 20 placentas reduced severity, some to the point where CHI was absent. In agreement with observations made by other groups, recurrence of CHI was not always associated with adverse outcome (9, 22). Bos et al. in their systematic review stated that incidence and recurrence of CHI may vary due to differing inclusion and exclusion criteria across studies (1). A controversial criterion noted was the exclusion of cases with chronic villitis or villitis of unknown etiology alongside CHI. As concurrent CHI and chronic villitis lesions have been reported in 25–47% of placentas (15, 23), those with combined lesions were not excluded here as they represented a similar and considerable proportion of cases. Of particular interest was the observation that the incidence of chronic villitis did not differ across pregnancies and therefore seemed unaffected by intervention within the participant group studied. In addition, though chronic villitis is itself associated with FGR (24), outcomes of pregnancies with concurrent CHI and chronic villitis were not significantly different, suggesting that chronic villitis in the presence of CHI may not have been a strong factor in adverse outcome.

## Conclusion

The data from this retrospective study suggest that including at least one of hydroxychloroquine or prednisolone in treatment regimen of pregnancies following diagnosis of CHI is effective at reducing placental lesion severity. In turn, decreased severity of CHI improves the likelihood of livebirth in subsequent pregnancies. From this, we propose that a standardized treatment protocol including aspirin, heparin, prednisolone and hydroxychloroquine may prove beneficial in the management of CHI. Studies into the efficacy of both hydroxychloroquine and prednisolone as well as other immunomodulatory agents used within the condition is extremely limited, and feasibility of randomized controlled trials in affected women is low given their poor obstetric history. To overcome this challenge, collaboration between centers specializing in the management of CHI is required to increase sample sizes to allow sufficient evaluation of treatment regimen.

## Data Availability Statement

The raw data supporting the conclusions of this article will be made available by the authors, without undue reservation.

## Ethics Statement

The studies involving human participants were reviewed and approved by NRES Committee North West—Greater Manchester West and NRES Committee London—City & East. The patients/participants provided their written informed consent to participate in this study.

## Author Contributions

CB, IC, and AH contributed toward the conception and design of the study. CB and CW organized the study database. CB, AH, and EC collected the data. CB performed the data analysis and wrote the manuscript. AH and IC assisted with data analysis and data presentation. GB, EC, CT, and AH assisted with manuscript proofreading and corrections. All authors contributed toward revision and approved the manuscript for submission.

## Funding

This study was funded by Tommy's the Baby Charity.

## Conflict of Interest

The authors declare that the research was conducted in the absence of any commercial or financial relationships that could be construed as a potential conflict of interest.

## Publisher's Note

All claims expressed in this article are solely those of the authors and do not necessarily represent those of their affiliated organizations, or those of the publisher, the editors and the reviewers. Any product that may be evaluated in this article, or claim that may be made by its manufacturer, is not guaranteed or endorsed by the publisher.
